# Supplementary material for: Assessing the basic knowledge and awareness of dengue fever prevention among migrant workers in Klang Valley, Malaysia
Source: PLoS One. 2024 Feb 1;19(2):e0297527. doi: 10.1371/journal.pone.0297527 (PMC10833505; doi:10.1371/journal.pone.0297527)
Supplement: S1 Table — The final models were selected by removing predictors from a global model sequentially until all predictors in the model met the preselected criterion (p<0.05) and all those outside did not. The marginal R2 value reflects the variance explained by fixed factors, whereas the conditional R2 represents the variance explained by both fixed and random factors [32]. (DOCX) [file pone.0297527.s001.docx]

**S1 Table. Final Regression Models.** The final models were selected by removing predictors from a global model sequentially until all predictors in the model met the preselected criterion (*p*<0.05) and all those outside did not. The marginal R^2^ value reflects the variance explained by fixed factors, whereas the conditional R^2^ represents the variance explained by both fixed and random factors ^32^.

| **Linear Regression Models** | **Marginal R^2^** | **Conditional R^2^** |
| --- | --- | --- |
| Knowledge ~ Age + Attitude + Practice + Education + Understand.SocialMedia + (1\|Nationality) | 0.109 | 0.508 |
| Attitude ~ Years + Knowledge + Responsibility + (1\|Nationality) | 0.095 | 0.515 |
| Practice ~ Knowledge + Responsibility + (1\|Nationality) | 0.048 | 0.474 |
