# Supplementary material for: Assessing the basic knowledge and awareness of dengue fever prevention among migrant workers in Klang Valley, Malaysia
Source: PLoS One. 2024 Feb 1;19(2):e0297527. doi: 10.1371/journal.pone.0297527 (PMC10833505; doi:10.1371/journal.pone.0297527)
Supplement: S2 Table — The Tucker–Lewis index (TLI) allows for comparisons between the proposed and null model whilst comparative fit index (CFI), more specifically, measures the improvement in non-centrality between the two models, with values>0.9 indicating good model fit [37]. The standardised root mean square residual (SRMR) assesses the extent to which the sample variance-covariance data fits the PSEM, with values<0.05 providing substantial support for the model [37]. (DOCX) [file pone.0297527.s002.docx]

**S2 Table. Post Hoc Analysis of PSEM Model.** The Tucker–Lewis index (TLI) allows for comparisons between the proposed and null model whilst comparative fit index (CFI), more specifically, measures the improvement in non-centrality between the two models, with values>0.9 indicating good model fit ^37^. The standardised root mean square residual (SRMR) assesses the extent to which the sample variance-covariance data fits the PSEM, with values<0.05 providing substantial support for the model ^37^.

| **Fit Indices** | **Model Value** | **Accepted Value** |
| --- | --- | --- |
| CFI | 0.974 | CFI > 0.9 |
| TLI | 0.933 | TLI > 0.9 |
| SRMR | 0.025 | SRMR < 0.05 |
