# Supplementary figures and images for: Assessing the basic knowledge and awareness of dengue fever prevention among migrant workers in Klang Valley, Malaysia
Source: PLoS One. 2024 Feb 1;19(2):e0297527. doi: 10.1371/journal.pone.0297527 (PMC10833505; doi:10.1371/journal.pone.0297527)

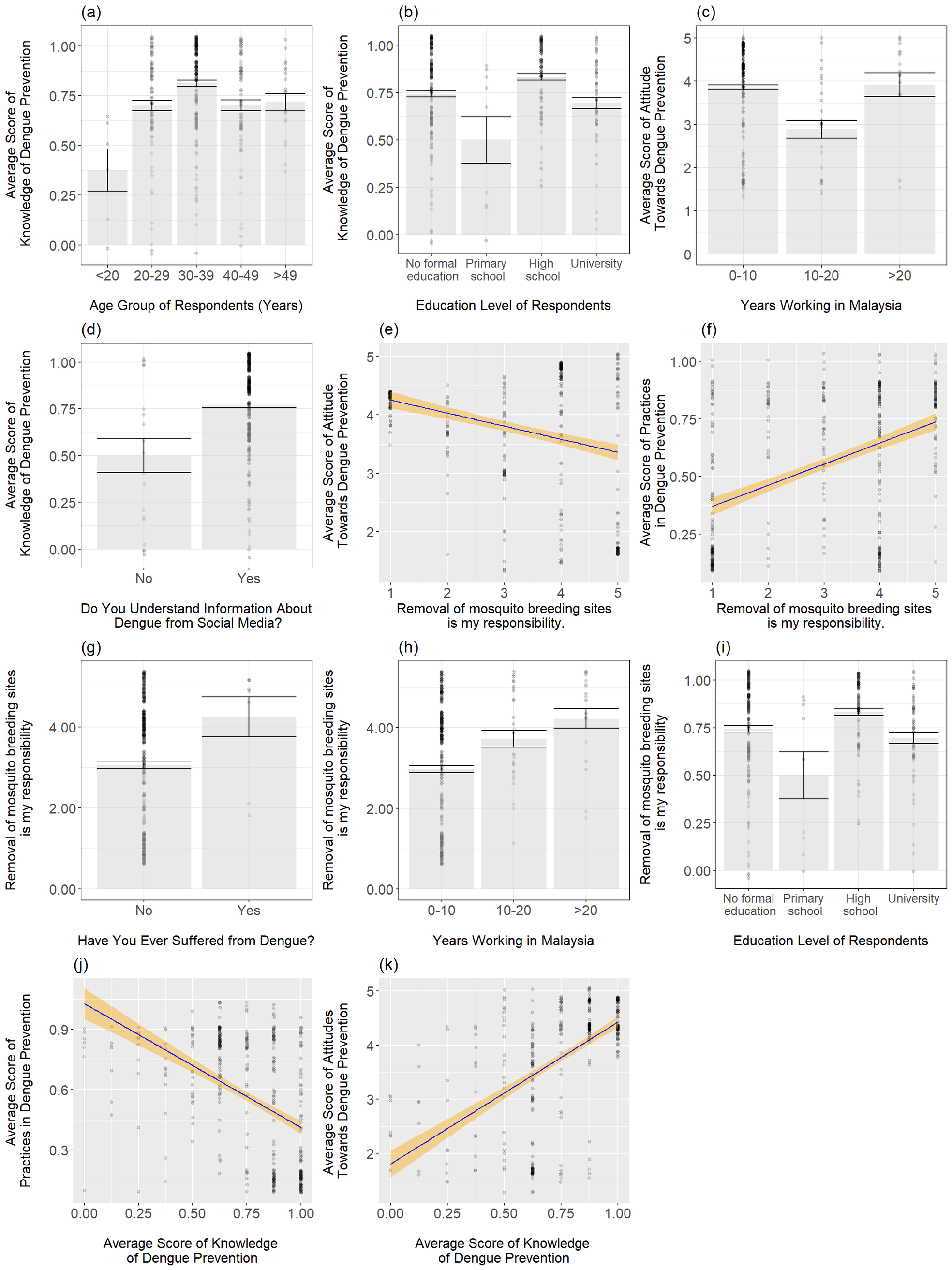

Supplement: S1 Fig — (a) ‘Practice’ plotted against ‘Knowledge’, (b) ‘Attitude’ plotted against ‘Knowledge’, (c) ‘Practice plotted against ‘Attitude’, (d) ‘Knowledge’ plotted against ‘Age’, (e) ‘Knowledge’ plotted against ‘Gender’, (f) ‘Knowledge’ plotted against ‘Years Working in Malaysia’, (g) ‘Knowledge’ plotted against ‘Understanding DF Information from Social Media’, (h) ‘Attitude’ plotted against ‘Education’, (i) ‘Attitude’ plotted against ‘Gender’, (j) ‘Practice’ plotted against ‘Gender’, (k) ‘Practice’ plotted against ‘Understanding DF Information from Social Media’. Line of best fit (blue) with 90% confidence interval (orange) was plotted for (a), (b) and (c). Responses were superimposed on predicted group mean (grey) and standard error of means (blue bar) for (d), (e), (f), (g), (h), (i), (j) and (k). (TIF) [file pone.0297527.s003.tif]
