# Supplementary material for: Assessing the basic knowledge and awareness of dengue fever prevention among migrant workers in Klang Valley, Malaysia
Source: PLoS One. 2024 Feb 1;19(2):e0297527. doi: 10.1371/journal.pone.0297527 (PMC10833505; doi:10.1371/journal.pone.0297527)
Supplement: S1 File — (ZIP) [file pone.0297527.s004.zip › Questionnaire_English.pdf]

Knowledge, Attitude and Practices on Common Infectious Diseases among Migrant Workers in Malaysia

Investigator:
Dr. Norhidayu Sahimin
Senior Lecturer
Tropical Infectious Diseases Research and Education Centre (TIDREC), Universiti Malaya, 50603 Kuala Lumpur, Malaysia
Email: ayusahimin@um.edu.my
Tel: +60123639245

Dr. Adzzie Shazleen Azman
Lecturer
School of Science, Monash University Malaysia, 47500 Bandar Sunway Selangor Darul Ehsan
Email: adzzieshazleen.azman@monash.edu
Tel: +60137225486

Introduction: The following information is provided for you to decide whether you wish to participate in this study on multifaceted impacts of communicable and non-communicable diseases among the vulnerable urban communities in Malaysia: inequities and co-benefits of interventions. You should feel free to ask any questions that you might have. You should also be aware that you can choose to withdraw from this study at any time.

Purpose: The purpose of this study is to contribute to a better understanding of the multifaceted impacts of a variety of diseases among the vulnerable low-income urban communities in Malaysia and to understand their knowledge, attitude and practices towards certain common infectious diseases in Malaysia

Study Procedure: Data collection occurs via a mobile application. The survey will be uploaded in the online platform to reduce the risk of transmission of Covid-19. It consists of a set of questionnaires which usually takes less than 30 minutes to complete. The questionnaires ask about your socio-demography information such as nationality, gender, age and employment status.

Participation in the Study: Participation in this study is completely voluntary. You are free to decline to participate, to end participation at any time for any reason, or to refuse to answer any individual question without penalty. Your decision whether to participate or not will not affect your relationship with this university and the services it may provide to you.

Benefit of Study: There is no direct benefit to participants. If you have any questions about this study or your rights, please contact the investigator, Dr. Norhidayu Sahimin at telephone number +60123639245 or email ayusahimin@um.edu.my.

Risk of study: We believe participation in this study is of minimal risk. However, if you ever feel uncomfortable during the study, you are free to stop at any time. As with any study that collects information about you, there is possible risk of loss of confidentiality. However, as described below, we have taken several measures to help prevent this.

Confidentiality: All of the data we collect will be held in confidence, because keeping your information confidential is extremely important to us. We plan to publish the information from this study in journals, and we also plan to present our findings in academic and research settings. In anything we write or present, we will use unique codes, diligent effort will be made to preserve the anonymity of participants. A copy of this consent statement is being provided for you to keep.

All the information from this study will be stored securely. All the data collected will be stored on a secure, HIPAA-compliant online storage account. All paper documents will be uploaded and stored to this account, then safely disposed of.

Complaints
Should you have any concerns or questions about this research project, which you do not wish to discuss with the researchers listed in this document, then you may contact:

Reviewing REC: University of Malaya Research Ethics Committee (UMREC)
Telephone: 03-79677022 (ext : 2369)
Email: umrec@um.edu.my
Mailing address: Pusat Perkhidmatan Penyelidikan (PPP), Level 2, Kompleks Pengurusan Penyelidikan dan Inovasi (KPPI), Universiti Malaya, 50603 Kuala Lumpur, Malaysia

* Indicates required question

1. By clicking “I agree” below you are indicating that you are at least 18 years old, have read and understood this consent form and agree to participate in this research study. *

Mark only one oval.

- Agree
- Disagree

Part A: Socio-demographic Profile

2. Sex *

Mark only one oval.

- Male
- Female

3. Age *

4. Date of birth *

Example: January 7, 2019

5. Nationality *

6. Level of Education *

Mark only one oval.

- University
- High school
- Primary school
- No formal education

7. District of residence *

Mark only one oval.

- Kuala Lumpur
- Gombak
- Hulu Langat
- Kuala Langat
- Sepang
- Petaling
- Klang
- Hulu Selangor
- Sabak Bernam

8. Since when have you been working in Malaysia? *

Example: January 7, 2019

9. Current Occupation Sectors *

Mark only one oval.

- Domestic helpers
- Construction
- Manufacturing
- Services
- Plantation
- Agriculture

Part F: Knowledge, attitude and practices on dengue fever

10. Tick (/) the answer of your choice based on your current knowledge *

Mark only one oval per row.

	True	False	I am not sure
**Dengue fever is caused by mosquitoes.**			
**All mosquitoes found in our environment carry the dengue virus.**			
**Only female mosquitoes suck blood.**			
**Mosquitoes lay their eggs in stagnant and dirty water.**			
**Mosquitoes are actively bite in the afternoon.**			
**Dengue fever can spread among people through mosquito bites.**			
**Symptoms of dengue fever include fever, joint pain and rash.**			
**Dengue fever can be cured**			

only by taking paracetamol.			
Dengue fever can be prevented by eliminating mosquito breeding grounds.			
Wearing clothing with bright colour that covers the body is a step taken to prevent mosquito bites.			
Mosquito bites can be avoided by using an insect repellent lotion/ liquid/spray and mosquito net			

11. Tick (/) one attitude of your choice with a selection from 1 (strongly disagree) to 5 (strongly agree). *

Mark only one oval per row.

	1 Strongly disagree	2 Disagree	3 Neutral	4 Agree	5 Strongly Agree
**Dengue fever is very dangerous and can cause fatal.**	◯	◯	◯	◯	◯
**I am at risk to get infected with dengue fever.**	◯	◯	◯	◯	◯
**Following all prevention control can prevent myself from getting the dengue fever.**	◯	◯	◯	◯	◯
**Removal of mosquito breeding sites will reduce the chance of dengue fever infection.**	◯	◯	◯	◯	◯
**Removal of mosquito breeding sites is not my responsibility.**	◯	◯	◯	◯	◯
**I will take part in a public activity for dengue control.**	◯	◯	◯	◯	◯

12. Tick (/) one answer of your choice for the practices listed below; usually (most of the time), sometimes (rarely) or never. *

Mark only one oval per row.

	Usually	Sometimes	Never
**Have you ever eliminated mosquito breeding grounds?**	◯	◯	◯
**Have you ever seen larvae in your residential / working area?**	◯	◯	◯
**Do you regularly use insecticide sprayers to kill mosquitoes?**	◯	◯	◯
**Do you use insect repellent creams / liquids/spray while outdoors?**	◯	◯	◯
**Do you wear covered, brightly colored clothing while doing outdoor activities?**	◯	◯	◯
**Do you install mosquito nets during sleep?**	◯	◯	◯

Have you ever take part in public activity for dengue control?			

Have you ever take part in public activity for dengue control?

13. Tick (/) the answer of your choice based on the access to the information on dengue fever. Do you get the information on dengue fever disease from: *

Mark only one oval per row.

	Yes	No
**Embassy/ Home Country Government**		
**Supervisor or other staff at work place/ Colleagues/ Friends**		
**Social Media (Television/ Facebook/ etc)**		
**Posters/ Billboards**		

14. Tick (/) the answer of your choice based on your understanding to the information on dengue fever. Do you understand the information of dengue fever disease from: *

Mark only one oval per row.

	Yes	No
**Embassy/ Home Country Government**	⬭	⬭
**Supervisor or other staff at work place/ Colleagues/ Friends**	⬭	⬭
**Social Media (Television/ Facebook/ etc)**	⬭	⬭
**Posters/ Billboards**	⬭	⬭

This content is neither created nor endorsed by Google.

Google Forms
