# Supplementary material for: Assessing the basic knowledge and awareness of dengue fever prevention among migrant workers in Klang Valley, Malaysia
Source: PLoS One. 2024 Feb 1;19(2):e0297527. doi: 10.1371/journal.pone.0297527 (PMC10833505; doi:10.1371/journal.pone.0297527)
Supplement: S1 File — (ZIP) [file pone.0297527.s004.zip › Questionnaire_Malay.pdf]

Pengetahuan, Sikap dan Praktik Tentang Infeksi Umum di Kalangan Pekerja Migran di Malaysia

Peneliti:

Dr. Norhidayu Sahimin
Pensyarah Kanan
Tropical Infectious Diseases Research and Education Centre (TIDREC), Universiti Malaya, 50603 Kuala Lumpur, Malaysia
Emel: ayusahimin@um.edu.my
Tel: +60123639245

Dr. Adzzie Shazleen Azman
Pensyarah
School of Science, Monash University Malaysia, 47500 Bandar Sunway
Selangor Darul Ehsan
Email: adzzieshazleen.azman@monash.edu
Tel: +60137225486

Pendahuluan: Informasi berikut ini disediakan bagi anda untuk memutuskan apakah anda ingin berpartisipasi dalam penelitian tentang dampak beragam penyakit menular dan tidak menular di antara komunitas perkotaan yang rentan di Malaysia: ketidakadilan dan manfaat tambahan dari intervensi. Anda bebas untuk mengajukan pertanyaan apa pun yang Anda miliki. Anda juga dapat memilih untuk mengundurkan diri dari penelitian ini kapan saja.

Tujuan: Tujuan dari penelitian ini adalah untuk berkontribusi pada pemahaman yang lebih baik tentang dampak beragam dari berbagai penyakit di antara komunitas perkotaan berpenghasilan rendah yang rentan di Malaysia dan untuk memahami pengetahuan, sikap, dan praktik mereka terhadap penyakit menular umum tertentu di Malaysia

Prosedur penelitian: Pengumpulan data dilakukan melalui aplikasi seluler. Survei akan diunggah secara daring untuk mengurangi risiko penularan COVID-19. Survey ini terdiri dari satu set kuesioner yang biasanya membutuhkan waktu kurang dari 30 menit untuk menyelesaikannya. Kuesioner menanyakan tentang informasi sosio-demografi anda seperti warganegara, jenis kelamin, usia dan status pekerjaan.

Partisipasi dalam penelitian: Partisipasi dalam penelitian ini sepenuhnya bersifat sukarela. Anda bebas untuk menolak berpartisipasi, mengakhiri partisipasi kapan saja dengan alasan apa pun, atau menolak menjawab pertanyaan individu tanpa denda. Keputusan anda untuk berpartisipasi atau tidak, tidak akan memengaruhi hubungan Anda dengan universitas ini dan layanan yang mungkin diberikannya kepada anda.

Manfaat penelitian: Tidak ada manfaat secara langsung bagi peserta. Jika anda memiliki pertanyaan tentang penelitian ini atau hak Anda, silakan hubungi peneliti, Dr. Norhidayu Sahimin di nomor telepon +60123639245 atau email ayusahimin@um.edu.my.

Risiko penelitian: Kami yakin partisipasi dalam penelitian ini memiliki risiko yang rendah. Namun, jika anda merasa tidak nyaman selama penelitian, anda bebas untuk berhenti kapan saja. Seperti halnya penelitian apa pun yang mengumpulkan informasi tentang anda, ada kemungkinan risiko hilangnya kerahasiaan. Namun, seperti yang dijelaskan di bawah ini, kami telah mengambil beberapa langkah untuk membantu mencegah hal ini.

Kerahasiaan: Semua data yang kami kumpulkan akan kami jaga kerahasiaannya, karena menjaga kerahasiaan informasi Anda sangat penting bagi kami. Kami berencana untuk mempublikasikan informasi dari penelitian ini di jurnal, dan kami juga berencana untuk mempresentasikan temuan kami dalam bentuk seminar akademik dan penelitian. Apa pun yang kami tulis atau presentasikan, kami akan menggunakan kode unik, upaya keras akan dilakukan untuk menjaga anonimitas peserta. Salinan pernyataan persetujuan ini disediakan untuk Anda simpan.

Semua informasi dari penelitian ini akan disimpan dengan aman. Semua data yang dikumpulkan akan disimpan di akun penyimpanan online yang aman dan sesuai dengan HIPAA. Semua dokumen kertas akan diunggah dan disimpan di akun ini, kemudian dibuang dengan aman.

Keluhan

Jika Anda memiliki kekhawatiran atau pertanyaan tentang proyek penelitian ini, yang tidak ingin anda diskusikan dengan para peneliti yang tercantum dalam dokumen ini, maka anda dapat menghubungi:

Tinjauan REC: University of Malaya Research Ethics Committee (UMREC)
Telepon: 03-79677022 (ext : 2369)
Email: umrec@um.edu.my
Alamat email: Pusat Perkhidmatan Penyelidikan (PPP), Level 2, Kompleks Pengurusan Penyelidikan dan Inovasi (KPPI), Universiti Malaya, 50603 Kuala Lumpur, Malaysia.

* Indicates required question

1. Dengan mengklik “Saya setuju” di bawah ini, anda menunjukkan bahwa Anda berusia minimal 18 tahun, telah membaca dan memahami formulir persetujuan ini dan setuju untuk berpartisipasi dalam penelitian ini. *

Mark only one oval.

- ( ) Setuju
- ( ) Tidak setuju

Bahagian A: Data Pribadi

2. Jenis Kelamin *

Mark only one oval.

- Lelaki
- Perempuan

3. Umur *

4. Tanggal lahir *

Example: January 7, 2019

5. Warganegara *

6. Tingkat pendidikan *

Mark only one oval.

- Universitas
- SMA/ SMP
- Sekolah dasar
- Tidak ada pendidikan formal

7. Wilayah tempat tinggal *

Mark only one oval.

- Kuala Lumpur
- Gombak
- Hulu Langat
- Kuala Langat
- Sepang
- Petaling
- Klang
- Hulu Selangor
- Kuala Selangor
- Sabak Bernam

8. Sejak kapan anda bekerja di Malaysia? *

____

Example: January 7, 2019

9. Sektor pekerjaan saat ini? *

Mark only one oval.

- Pembantu rumah tangga
- Konstruksi
- Manufaktur
- Servis
- Perkebunan
- Pertanian

Pengetahuan, sikap dan praktik tentang demam berdarah dengue (DBD)

10. Centang (/) pilihan jawaban anda berdasarkan pengetahuan anda saat ini *

Mark only one oval per row.

	Benar	Salah	Tidak pasti
**Penyakit demam berdarah dengue disebabkan oleh nyamuk.**	⬭	⬭	⬭
**Semua nyamuk yang berada di lingkungan kita membawa virus dengue.**	⬭	⬭	⬭
**Hanya nyamuk betina yang menghisap darah.**	⬭	⬭	⬭
**Nyamuk bertelur di air yang menggenang dan kotor.**	⬭	⬭	⬭
**Nyamuk aktif menggigit pada sore hari.**	⬭	⬭	⬭
**Demam berdarah dengue dapat menyebar di masyarakat melalui gigitan nyamuk.**	⬭	⬭	⬭
**Gejala demam berdarah dengue termasuk demam, nyeri sendi dan ruam.**	⬭	⬭	⬭

Demam berdarah dengue bisa disembuhkan hanya dengan meminum parasetamol.
Demam berdarah dengue bisa disembuhkan hanya dengan meminum parasetamol.

Demam berdarah dengue dapat dicegah dengan menghilangkan tempat pembiakan nyamuk.
Demam berdarah dengue dapat dicegah dengan menghilangkan tempat pembiakan nyamuk.

Mengenakan pakaian dengan warna cerah yang menutupi tubuh merupakan langkah yang dilakukan untuk mencegah gigitan nyamuk.
Mengenakan pakaian dengan warna cerah yang menutupi tubuh merupakan langkah yang dilakukan untuk mencegah gigitan nyamuk.

Gigitan nyamuk dapat dihindari dengan menggunakan lotion anti serangga/ cairan/ semprot dan kelambu
Gigitan nyamuk dapat dihindari dengan menggunakan lotion anti serangga/ cairan/ semprot dan kelambu

11. Centang (/) salah satu sikap dari pilihan 1 (sangat tidak setuju) sampai 5 (sangat setuju). *

Mark only one oval per row.

	1 Sangat tidak setuju	2 Tidak setuju	3 Netral	4 Setuju	5 Sangat setuju
**Demam berdarah dengue sangat berbahaya dan bisa berakibat fatal.**	⬭	⬭	⬭	⬭	⬭
**Saya berisiko tertular penyakit demam berdarah dengue.**	⬭	⬭	⬭	⬭	⬭
**Mengikuti semua protokol pencegahan dapat mencegah diri saya terkena demam berdarah dengue.**	⬭	⬭	⬭	⬭	⬭
**Membasmi tempat perkembangbiakan nyamuk akan mengurangi kemungkinan infeksi demam berdarah dengue.**	⬭	⬭	⬭	⬭	⬭
**Pembasmian tempat perkembangbiakan nyamuk bukan tanggung jawab saya.**	⬭	⬭	⬭	⬭	⬭
**Saya akan berpartisipasi dalam kegiatan masyarakat untuk pengendalian demam berdarah dengue.**	⬭	⬭	⬭	⬭	⬭

12. Centang (/) salah satu praktik untuk kegiatan yang tercantum di bawah ini; Sering (selalu), Terkadang (jarang) atau tidak pernah. *

Mark only one oval per row.

	Sering	Terkadang	Tidak pernah
**Apakah anda pernah membasmi tempat perkembangbiakan nyamuk?**	⬭	⬭	⬭
**Pernahkah anda melihat jentik-jentik di lingkungan tempat tinggal/ kerja anda?**	⬭	⬭	⬭
**Apakah anda biasanya menggunakan penyemprot insektisida untuk membunuh nyamuk?**	⬭	⬭	⬭
**Apakah anda menggunakan krim/ cairan/semprotan anti serangga saat berada di luar ruangan?**	⬭	⬭	⬭
**Apakah anda mengenakan pakaian tertutup berwarna cerah saat melakukan aktivitas di luar ruangan?**	⬭	⬭	⬭
**Apakah anda memasang kelambu saat tidur?**	⬭	⬭	⬭
Apakah anda pernah mengikuti			

kegiatan masyarakat untuk pengendalian demam berdarah dengue?

Apakah anda pernah mengikuti kegiatan masyarakat untuk pengendalian demam berdarah dengue?			

13. Centang (/) pilihan jawaban anda berdasarkan akses anda terhadap informasi demam berdarah dengue. Apakah anda mendapatkan informasi tentang demam berdarah dengue dari: *

Mark only one oval per row.

	Iya	Tidak
**Kedutaan/ Pemerintah Negara Asal**		
**Pembimbing atau staf lain di tempat kerja/ Rekan kerja/ Teman**		
**Sosial Media (Televisi/ Facebook/ dll.)**		
**Poster/ Papan iklan**		

14. Centang (/) pilihan jawaban anda berdasarkan pemahaman anda terhadap informasi demam berdarah dengue. Apakah anda memahami informasi tentang demam berdarah dengue dari: *

Mark only one oval per row.

	Iya	Tidak
**Kedutaan/ Pemerintah Negara Asal**	⬭	⬭
**Pembimbing atau staf lain di tempat kerja/ Rekan kerja/ Teman**	⬭	⬭
**Sosial Media (Televisi/ Facebook/ dll.)**	⬭	⬭
**Poster/ Papan iklan**	⬭	⬭

This content is neither created nor endorsed by Google.

Google Forms
