# Supplementary material for: Assessing the basic knowledge and awareness of dengue fever prevention among migrant workers in Klang Valley, Malaysia
Source: PLoS One. 2024 Feb 1;19(2):e0297527. doi: 10.1371/journal.pone.0297527 (PMC10833505; doi:10.1371/journal.pone.0297527)
Supplement: S3 File — (PDF) [file pone.0297527.s006.pdf]

Data pribadi (RAHASIA)

**ID Kode**				
**Jenis kelamin**				
**Umur**				
**Tanggal lahir**				
**Kebangsaan**				
**Tingkat pendidikan**				
**Distrik tempat tinggal (Contoh: Klang)**	**Kuala Lumpur**		**Petaling**	
	**Gombak**		**Dentang**	
	**Thread Hulu**		**Hulu Selangor**	
	**Kuala Langat**		**Kuala Selangor**	
	**Sepang**		**Sabak Bernam**	
**Sejak kapan Anda berada di Malaysia? (tinggal saat ini)<br>(Contoh: Maret 2020)**				
**Apakah Anda tinggal di Malaysia untuk bekerja sebelumnya?**	Tahun: Durasi:			
**Pekerjaan saat ini**				
**Informasi Kesehatan**				
**Pernahkah Anda menderita COVID-19 seperti yang diceritakan oleh dokter atau profesional kesehatan? (Silakan kelilingi)**	**Ya / Tidak**			

Pernahkah Anda mengalami infeksi ini? Jika YA, harap lingkari tingkat keparahannya
1= Sering; 2=Langka; 3=Tidak pernah

**Berdarah**			**Demam tifoid**			**Kolera**			**Keracunan makanan**		
1	2	3	1	2	3	1	2	3	1	2	3

Demam berdarah

Centang (/) jawaban pilihan Anda berdasarkan pengetahuan Anda saat ini

	**Pengetahuan tentang penyakit demam berdarah**	**Benar**	**Palsu**	**Saya tidak yakin**
1	Demam berdarah disebabkan oleh nyamuk.			
2	Semua nyamuk yang ditemukan di lingkungan kita membawa virus dengue.			
3	Hanya nyamuk betina yang menghisap darah.			
4	Nyamuk bertelur di air yang tergenang dan kotor.			
5	Nyamuk aktif menggigit di sore hari.			
6	Demam berdarah dapat menyebar di antara orang-orang melalui gigitan nyamuk.			
7	Gejala demam berdarah termasuk demam, nyeri sendi dan ruam.			
8	Demam berdarah dapat disembuhkan hanya dengan mengonsumsi parasetamol.			
9	Demam berdarah dapat dicegah dengan menghilangkan tempat berkembang biak nyamuk.			
10	Mengenakan pakaian dengan warna cerah yang menutupi tubuh adalah langkah yang dilakukan untuk mencegah gigitan nyamuk.			
11	Gigitan nyamuk dapat dihindari dengan menggunakan lotion / cairan / semprotan obat nyamuk dan kelambu			

Centang (/) satu jawaban pilihan Anda dengan formulir pilihan 1 (sangat tidak setuju) hingga 5 (sangat setuju).

N.	**Sikap terhadap penyakit demam berdarah**	**1 Sangat tidak setuju**	**2 Tidak setuju**	**3 Netral**	**4 Setuju**	**5 Sangat setuju**
1	Demam berdarah sangat berbahaya dan dapat menyebabkan kematian.					
2	Saya berisiko terinfeksi demam berdarah.					
3	Mengikuti semua kontrol pencegahan dapat mencegah diri dari mendapatkan demam berdarah.					
4	Penghapusan tempat perkembangbiakan nyamuk akan mengurangi kemungkinan infeksi demam berdarah.					
5	Penghapusan tempat perkembangbiakan nyamuk bukan tanggung jawab saya.					
6	Saya akan mengambil bagian dalam kegiatan publik untuk pengendalian demam berdarah.					

Centang (/) satu jawaban pilihan Anda untuk praktik yang tercantum di bawah ini; Biasanya (selalu), terkadang (jarang) atau tidak pernah.

M.	**Praktik penyakit demam berdarah**	**Biasanya**	**Kadang-kadang**	**Tidak pernah**
1	Pernahkah Anda menghilangkan tempat berkembang biak nyamuk?			
2	Pernahkah Anda melihat larva di area perumahan / kerja Anda?			
3	Apakah Anda secara teratur menggunakan penyemprot insektisida untuk membunuh nyamuk?			
4	Apakah Anda menggunakan krim / cairan / semprotan pengusir serangga saat berada di luar ruangan?			
5	Apakah Anda mengenakan pakaian tertutup berwarna cerah saat melakukan aktivitas di luar ruangan?			
6	Apakah Anda memasang kelambu saat tidur?			
7	Pernahkah Anda mengambil bagian dalam kegiatan publik untuk pengendalian demam berdarah?			

Centang (/) jawaban pilihan Anda berdasarkan akses dan pemahaman Anda terhadap informasi tentang demam berdarah

O	**Akses ke informasi tentang Demam Berdarah**	**Ya**	**Tidak**
1	Apakah Anda mendapatkan informasi tentang penyakit demam berdarah dari:		
	a) Kedutaan Besar / Pemerintah Negara Asal		
	b) Supervisor atau staf lain di tempat kerja / Kolega / Teman		
	c) Media Sosial (Televisi / Facebook / dll)		
	d) Poster/Baliho		
2	Apakah Anda memahami informasi penyakit demam berdarah dari:		
	a) Kedutaan Besar / Pemerintah Negara Asal		
	b) Supervisor atau staf lain di tempat kerja / Kolega / Teman		
	c) Media Sosial (Televisi / Facebook / dll)		
	d) Poster/Baliho		

Terima kasih telah menjawab survei.
