# Supplementary material for: Assessing the basic knowledge and awareness of dengue fever prevention among migrant workers in Klang Valley, Malaysia
Source: PLoS One. 2024 Feb 1;19(2):e0297527. doi: 10.1371/journal.pone.0297527 (PMC10833505; doi:10.1371/journal.pone.0297527)
Supplement: S4 File — (PDF) [file pone.0297527.s007.pdf]

Personal details (CONFIDENTIAL)

<table>
<tr><td>Code ID</td><td colspan="12"></td></tr>
<tr><td>Sex</td><td colspan="12"></td></tr>
<tr><td>Age</td><td colspan="12"></td></tr>
<tr><td>Date of birth</td><td colspan="12"></td></tr>
<tr><td>Nationality</td><td colspan="12"></td></tr>
<tr><td>Level of education</td><td colspan="12"></td></tr>
<tr><td rowspan="5">District of residence (Example: Klang)</td><td colspan="3">Kuala Lumpur</td><td colspan="3"></td><td colspan="3">Petaling</td><td colspan="3"></td></tr>
<tr><td colspan="3">Gombak</td><td colspan="3"></td><td colspan="3">Klang</td><td colspan="3"></td></tr>
<tr><td colspan="3">Hulu Langat</td><td colspan="3"></td><td colspan="3">Hulu Selangor</td><td colspan="3"></td></tr>
<tr><td colspan="3">Kuala Langat</td><td colspan="3"></td><td colspan="3">Kuala Selangor</td><td colspan="3"></td></tr>
<tr><td colspan="3">Sepang</td><td colspan="3"></td><td colspan="3">Sabak Bernam</td><td colspan="3"></td></tr>
<tr><td>Since when have you been in Malaysia? (current stay) (Example: March 2020)</td><td colspan="12"></td></tr>
<tr><td>Did you stay in Malaysia for work previously?</td><td colspan="6">Year:</td><td colspan="6">Duration:</td></tr>
<tr><td>Current occupation</td><td colspan="12"></td></tr>
<tr><td colspan="13">Health Information</td></tr>
<tr><td>Have you ever had COVID-19 as told by the doctor or health professionals? (Please encircle)</td><td colspan="12">Yes / No</td></tr>
<tr><td colspan="13">Have you ever had these infections? If YES, please encircle the severity
1= Often; 2=Rare; 3=Never</td></tr>
<tr><td colspan="3">Dengue</td><td colspan="3">Typhoid fever</td><td colspan="3">Cholera</td><td colspan="3">Food poisoning</td></tr>
<tr><td>1</td><td>2</td><td>3</td><td>1</td><td>2</td><td>3</td><td>1</td><td>2</td><td>3</td><td>1</td><td>2</td><td>3</td></tr>
</table>

Dengue Fever

Tick (/) the answer of your choice based on your current knowledge

M.	**Knowledge on dengue fever disease**	**True**	**False**	**I'm not sure**
1	Dengue fever is caused by mosquitoes.			
2	All mosquitoes found in our environment carry the dengue virus.			
3	Only female mosquitoes suck blood.			
4	Mosquitoes lay their eggs in stagnant and dirty water.			
5	Mosquitoes are actively bite in the afternoon.			
6	Dengue fever can spread among people through mosquito bites.			
7	Symptoms of dengue fever include fever, joint pain and rash.			
8	Dengue fever can be cured only by taking paracetamol.			
9	Dengue fever can be prevented by eliminating mosquito breeding grounds.			
10	Wearing clothing with bright colour that covers the body is a step taken to prevent mosquito bites.			
11	Mosquito bites can be avoided by using an insect repellent lotion/ liquid/spray and mosquito net			

Tick (/) one answer of your choice with a selection form 1 (strongly disagree) to 5 (strongly agree).

N.	**Attitudes on dengue fever disease**	**1 Strongly disagree**	**2 Disagree**	**3 Neutral**	**4 Agree**	**5 Strongly agree**
1	Dengue fever is very dangerous and can cause fatal.					
2	I am at risk to get infected with dengue fever.					
3	Following all prevention control can prevent myself from getting the dengue fever.					
4	Removal of mosquito breeding sites will reduce the chance of dengue fever infection.					
5	Removal of mosquito breeding sites is not my responsibility.					
6	I will take part in a public activity for dengue control.					

Tick (/) one answer of your choice for the practices listed below; usually (always), sometimes (rarely) or never.

M.	**Practices on dengue fever disease**	**Usually**	**Sometimes**	**Never**
1	Have you ever eliminated mosquito breeding grounds?			
2	Have you ever seen larvae in your residential / working area?			
3	Do you regularly use insecticide sprayers to kill mosquitoes?			
4	Do you use insect repellent creams / liquids/spray while outdoors?			
5	Do you wear covered, brightly colored clothing while doing outdoor activities?			
6	Do you install mosquito nets during sleep?			
7	Have you ever take part in public activity for dengue control?			

Tick (/) the answer of your choice based on the access and your understanding to the information on dengue fever

O	**Access to information on Dengue Fever**	**Yes**	**No**
1	Do you get the information on dengue fever disease from:		
	a) Embassy/ Home Country Government		
	b) Supervisor or other staff at work place/ Colleagues/ Friends		
	c) Social Media (Television/ Facebook/ etc)		
	d) Posters/ Billboards		
2	Do you understand the information of dengue fever disease from:		
	a) Embassy/ Home Country Government		
	b) Supervisor or other staff at work place/ Colleagues/ Friends		
	c) Social Media (Television/ Facebook/ etc)		
	d) Posters/ Billboards		

Thank you for your answering the survey.
