# Supplementary material for: Assessing the basic knowledge and awareness of dengue fever prevention among migrant workers in Klang Valley, Malaysia
Source: PLoS One. 2024 Feb 1;19(2):e0297527. doi: 10.1371/journal.pone.0297527 (PMC10833505; doi:10.1371/journal.pone.0297527)
Supplement: Attachment — Submitted filename: Response to Reviewers.docx [file pone.0297527.s009.docx]

**RESPONSE TO REVIEWERS**

Dear Academic Editor and Reviewers,

Thank you for your comments; we appreciate the time and effort taken to review our manuscript. Please see our responses to your comments below.

**General Comments:**

1. Please ensure that your manuscript meets PLOS ONE's style requirements, including those for file naming.

*Manuscript has been revised to meet PLOS ONE’s style requirements.*

2. We note that you have indicated that data from this study are available upon request. PLOS only allows data to be available upon request if there are legal or ethical restrictions on sharing data publicly. If there are no ethical or legal restrictions, please upload the minimal anonymized data set necessary to replicate your study findings as either Supporting Information files or to a stable, public repository and provide us with the relevant URLs, DOIs, or accession numbers. For a list of acceptable repositories, please see <http://journals.plos.org/plosone/s/data-availability#loc-recommended-repositories>.

*Data has been made available as Supplementary File 2.*

**Reviewer #1**

3. “A pilot study involving five Indonesians”, the pilot study should run at least on 30 participants not 5. ‘The internal consistency of the KAP questions/statements in the questionnaire was assessed using Cronbach’s Alpha test’, this test mostly used in pilot sample which contain at least 30 participant, if the result more than 0.7, then run the questionnaire to all participant in the study.

*The following sentence has been modified for clarity (Page 4, Line 91): ‘A pilot study was conducted to test the comprehension of the questionnaire wherein five study leaders distributed the questionnaire to ~10 respondents each.’*

*A Cronbach’s alpha value of ≥0.7 was used to indicate acceptable internal consistency of the questions to represent a single construct. Statements were removed from or retained in the final construct on this basis. We followed the methodology of Reference 24.*

4. There are multiple details and repeated data in data analysis. Please revise it and remove unnecessary data.

*The manuscript has been edited accordingly.*

5. You write: Dengue fever is fatal. Although Approximately 10% of dengue cases progress to severe disease, and the majority of cases have mild disease.

*Sentence has been rephrased to (Page 3, Line 57) ‘DF can be fatal, with symptoms including high fever, body pains and persistent vomiting’.*

6. Following the manufacturing industry (36%), most migrant workers in Malaysia are employed in the construction industry (19%). This sentence has something wrong, please revise it.

*Sentence has been rephrased to (Page 3, Line 71) ‘Most migrant workers in Malaysia are employed in the manufacturing (36%) and construction industry (19%)’.*

7. Write the table or the figure numbers at the end of the sentences.

*Table and figure numbers have been listed at the end of each sentence. There are a few exceptions to ensure the reader understands which figure/table to refer to for each component of the sentence.*

*E.g., ‘The final PSEM model (i) based on the results of the linear mixed effects modelling (S1 Table and S1 Fig), (ii) with lowest AIC value (71.62), (iii) p>0.05 for all directed separation tests and Fisher’s C test (C(30)=31.62, p=0.386), and (iv) fitted the data well in post-hoc assessments (S2 Table) was retained and visualised (Fig 3).’*

8. Multiple repeated the name of the test in the results section is not advisable as for example ; Dunn’s test , please remove it.

*The manuscript has been edited accordingly.*

9. References N 3, 8, 10, 23, 25, 26, 33 from website, please write it correctly with access day, month, year, and URL link.

*All references have been updated accordingly.*

10. Refrences N. 7, 11, 12, 14, 15, 21,39, 43 please write the pages numbers.

*All references have been updated accordingly.*

**Reviewer #2**

11. The manuscript was submitted without numbering the lines of the text, which makes it difficult to reference them in the review.

*The manuscript has been edited accordingly.*

12. In the first sentence of the Abstract, please indicate whether "390 million Dengue virus infetions" is a global or local prevalence. It is indicated in the introduction section, but please do the same for the abstract.

*Sentence has been rephrased to (Page 2, Line 34) ‘Globally, 390 million dengue virus infections occur per year’.*

13. In the line 6 of the last paragraph of introduction, "(i) better understand the level of and relationship between KAP" - Maybe it is better to not abbreviate KAP here but rather write it in full. It makes it easier to understand the sentence.

*The manuscript has been edited accordingly.*

14. Why did you choose to use 455 study participants? please explain the reason for choosing this sample size. Was it based on some sample size estimation method?

*The following sentence has been added for clarity (Page 5, Line 120): ‘The Krejcie and Morgan formula was applied, with a 5% margin of error and 95% confidence interval, resulting in a minimum sample size of 385 participants.’*

15. Please explain the Inclusion and Exclusion criteria for selecting participants.

*The Inclusion and Exclusion criteria was stated as follows (Page 5, Line 114): ‘Respondents from the Philippines and Nepal were invited for this study due to past collaboration with their respective coordinators, whereas Indonesian respondents were invited as they constitute the majority of migrant workers in Malaysia’.*

16. Please explain the nature of the variables used in the analysis. Were the answers converted to some number scale? are these categorical variables? What is the range of their values. This will help readers understand the reason for your choice of statistical analytical methods.

*The following sentence has been added for clarity (Page 5, Line 144): ‘The categorical explanatory variables were converted to numeric binary (e.g. 0 = male and 1 = female) or ordinal (e.g., 1 = no formal education, 2 = primary school, 3 = high school, 4 = university)’.*

17. In the selection of explanatory variables for analysis, please explain which variables were initially considered, how and why you eliminated some variables and what were the final variables included in the model. You provided very little explanation of the analysis.

*Explanatory variables that were initially considered were stated as follows (Page 5, Line 140): ‘The explanatory variables were ‘Gender’, ‘Age’, ‘Years Working in Malaysia’, ‘Education’, ‘Occupation’, ‘Dengue History’, ‘Understanding of DF Information from Social Media’ and ‘Responsibility’’.*

*Basis for variable elimination was stated as follows (Page 6, Line 146): ‘The final models were selected by removing predictors from a global model sequentially until all predictors in the model met the preselected criterion (p<0.05) and all those outside did not’.*

*The final variables included in the model are discussed in the Results (Pages 8-9 ) and Discussion sections. The model with the final selected variables is visualised in Figure 3.*
