# Supplementary material for: Assessing the basic knowledge and awareness of dengue fever prevention among migrant workers in Klang Valley, Malaysia
Source: PLoS One. 2024 Feb 1;19(2):e0297527. doi: 10.1371/journal.pone.0297527 (PMC10833505; doi:10.1371/journal.pone.0297527)
Supplement: Attachment — Submitted filename: R2-Response to Reviewers.docx [file pone.0297527.s010.docx]

**RESPONSE TO REVIEWERS**

Dear Academic Editor and Reviewers,

Thank you for your comments; we appreciate the time and effort taken to review our manuscript. Please see our responses to your comments below.

**General Comments:**

1. Please review your reference list to ensure that it is complete and correct. If you have cited papers that have been retracted, please include the rationale for doing so in the manuscript text, or remove these references and replace them with relevant current references. Any changes to the reference list should be mentioned in the rebuttal letter that accompanies your revised manuscript. If you need to cite a retracted article, indicate the article’s retracted status in the References list and also include a citation and full reference for the retraction notice.

*All cited references are correct and still available online, to the best of our knowledge.*

**Reviewer #3**

2. Is there any validity test on different versions of questionnaire used in the data collection, as you mention particularly Indonesian workers used Indonesian instead of English. Will this become a potential bias in methodology? Please elaborate your answer, and if there is a risk of bias or skewness arise, also add your reasonings in including the data.

*Tests for validity are discussed in detail as follows (Page 5, Line 126): ‘The internal consistency of the KAP questions/statements in the questionnaire was assessed using Cronbach’s Alpha test, using ‘alpha()’ from the ‘psych’ package ^26^. A Cronbach’s alpha value of ≥0.7 indicates acceptable internal consistency of the questions to represent a single construct ^27-29^.’*

*Data collection differed between the three nationalities (Page 5, Line 116): ‘Filipino respondents completed the questionnaire in English. Due to limited English proficiency among Nepali and Indonesian respondents, Nepali respondents underwent a phone interview conducted by their coordinator in their native language, whereas Indonesian respondents completed the questionnaire in the Indonesian language.’*

*To mitigate the effects of any potential bias, the following measure was taken (Page 5, Line 143): ‘Nationality’ was coded as a random error as the method of response collection varied between nationalities (e.g., English/Indonesian questionnaire, and phone interview).*

3. I would suggest for you to include individual analysis of each factor on KAP, instead of the final model stated in the results section. As also mentioned by the previous reviewer concerning the lack of details in selecting factors for final model, I believe your explanation can be added instead to the text.

*The individual linear regression models for each of knowledge, attitude and practices are included in the Appendix (S1 Table). The final, piecewise structural equation model was based on the aforementioned individual regression analyses. Piecewise structural equation modelling can accurately gauge complex multivariate relationships, whilst allowing variables to serve as both predictors and responses.*

*Details for selecting factors are included in the text as follows:*

- *Explanatory variables that were initially considered (Page 5, Line 140): ‘The explanatory variables were ‘Gender’, ‘Age’, ‘Years Working in Malaysia’, ‘Education’, ‘Occupation’, ‘Dengue History’, ‘Understanding of DF Information from Social Media’ and ‘Responsibility’’.*
- *Basis for variable elimination (Page 6, Line 146): ‘The final models were selected by removing predictors from a global model sequentially until all predictors in the model met the preselected criterion (p<0.05) and all those outside did not’.*

4. On discussion, please add further details and discussion on the results, especially the correlation between KAP and previously mentioned factors. I am personally intrigued by the negative correlation of knowledge and practice. Do you have any explanation or possible reasoning for such things?

*Each relationship depicted in Figure 3 is explored in the discussion section as follows:*

- *Correlation between knowledge, attitudes and practices under ‘*Dengue Prevention KAP*’ (Page 9, Line 236)*
- *Correlation between KAP and sociodemographic variables under ‘*Targeted Groups for Training Based on Socio-Demographic Analyses’ *(Page 10, Line 263)*
- *Correlation between KAP and social media under ‘*Social Media as a Tool for DF Awareness*’ (Page 11, 281)*

*The negative correlation between knowledge and practice was explored as follows (Page 10, Line 245):*

*‘Respondents seldom exercised DF preventive practices (56.5%). Knowledge and practices in DF prevention were negatively associated. Studies in Malaysia ^40-43^, Jamaica ^44^ and Thailand ^45^ also found that DF prevention knowledge does not necessarily translate into preventative practices. In migrant workers, this may be attributed to an inability to understand region-specific DF awareness campaigns due to limited proficiency in the local language ^17, 46^ and inability to afford precautionary measures (e.g., mosquito nets, insect repellent) due to low income ^44^. Moreover, our study found that preventative measures were frequently practised by those who considered removing mosquito breeding sites as their own responsibility, who were, in turn, those with previous history of DF, similar to past studies ^16 47^. This suggests a lack of motivation to change current behaviour/habits, unless individuals experience and fully perceive the risks of DF ^18, 41, 45, 48^.*
